# The Effect of Protein Tagging on Aggregation and Phase Separation

**DOI:** 10.1002/jcb.70096

**Published:** 2026-06-14

**Authors:** Harunobu Saito, Kenji Sugase

**Affiliations:** ^1^ Division of Applied Life Sciences, Graduate School of Agriculture Kyoto University, Kitashirakawa Oiwake‐cho, Sakyo‐ku Kyoto Japan

**Keywords:** aggregation, amyloid fibrils, biomolecular condensates, intrinsically disordered proteins, LLPS, protein tagging

## Abstract

Protein tags are widely used for purification, solubilization, detection, and imaging, yet they can substantially alter protein self‐assembly. This interference is particularly significant for intrinsically disordered proteins and low‐complexity domains, whose aggregation and phase separation are mediated by weak multivalent interactions that are easily disrupted by exogenous elements. In this review, we examine how affinity tags, solubility tags, fluorescent proteins, and chemical labels influence aggregation, amyloid formation, and liquid‐liquid phase separation (LLPS). We first classify recurring perturbation mechanisms into six primary categories: solubility enhancement, artificial multivalency, electrostatic interactions, local effects, metal coordination, and positional dependence. Crucially, these non‐exclusive mechanisms often operate simultaneously within a single construct. We then review representative case studies across pathogenic amyloids, RNA‐binding proteins, viral inclusions, functional amyloids, yeast prions, and membrane proteins. These examples demonstrate that tags alter assembly kinetics, phase boundaries, material properties, fibril morphology, oligomeric states, and observed phenotypes, rather than merely serving as neutral tools for detection. In some systems, tags suppress intrinsic assembly; in others, they promote non‐native condensation or stabilize alternative aggregate states. Finally, we discuss practical experimental strategies to distinguish intrinsic protein behavior from construct‐dependent effects, emphasizing matched comparisons, orthogonal validation, and the careful interpretation of measurements based on tag cleavage or fluorescence. Collectively, the evidence indicates that protein tags should be treated as experimental variables that shape assembly states rather than as inert technical additions.

## 1 Introduction

Understanding protein aggregation and condensation has become a central challenge in cellular biochemistry. Liquid‐liquid phase separation (LLPS) drives the assembly of biomolecular condensates, which achieve cellular compartmentalization and regulate biochemical reactions [1, 2, 3], while the formation of amyloid fibrils is directly linked to the pathogenesis of numerous neurodegenerative diseases [4, 5, 6]. Deciphering such mechanisms extensively relies on in vitro assays using purified proteins and *in vivo* assessments of cellular localization and function. These assembly and aggregation processes are frequently driven by intrinsically disordered proteins (IDPs), especially those harboring low‐complexity domains (LCDs) [7, 8]. Their behavior is dictated by the sum of weak, multivalent interactions, including hydrophobic, aromatic, and electrostatic forces [9, 10, 11]. Consequently, even minor perturbations to the interaction balance by exogenous elements can substantially alter experimental results, such as phase diagrams (e.g., saturation concentrations), nucleation and growth kinetics, droplet material properties (including hardening and gelation), and final aggregate morphologies.

**Figure 1:**
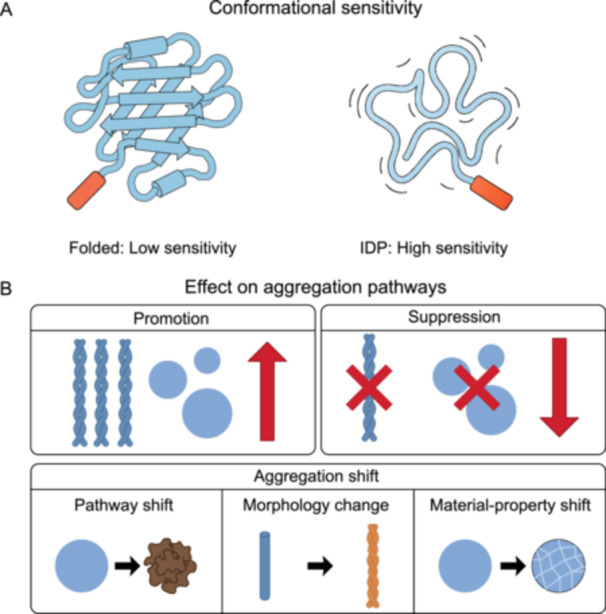
Conceptual overview of tag‐induced effects on protein assembly. (A) Folded proteins are often less sensitive to exogenous modifications than IDPs, whose assembly depends on weak multivalent interactions distributed across the sequence. (B) Tags, linkers, and labels can promote, suppress, or redirect aggregation and LLPS, and can alter the aggregation pathway, morphology, and material properties.

In practice, the addition of tags, linkers, fluorescent proteins, or chemical labels is practically unavoidable for protein purification, detection, and visualization. Common examples include affinity tags for purification, fusion proteins for solubility enhancement, fluorescent tags for cellular tracking, and chemical fluorophores for specific or random labeling. However, these exogenous additions introduce non‐native hydrophobicity, charges, steric bulk, or multivalency, thereby acting as active drivers that rewire the intrinsic interaction networks of the host proteins [12, 13]. For proteins with stable tertiary structures, the addition of a tag may leave the core fold largely unperturbed [14]. In contrast, because the entire sequence of an IDP can participate in intermolecular interactions [10, 11], the physical properties and insertion sites of tags, linkers, or labels often dictate the resulting assembly states. Beyond merely promoting or suppressing aggregation, these modifications can shift the specific type, morphology, and material properties of the assemblies. For instance, they can alter the balance between fibrillar and amorphous aggregates, change fibril structures, or accelerate the transition of liquid droplets to solid‐like states [15, 16] (Figure 1). Furthermore, the tag‐induced effects propagate to physiological behaviors in vivo, affecting cellular localization, condensation propensity, toxicity, and function [13, 17].

Because many existing experimental systems are not designed with tag‐induced effects in mind, they often fail to capture the intrinsic properties of the untagged proteins. When it remains unclear whether an observed phenomenon originates from the target protein itself or from the specific construct design, the resulting conclusions lose generalizability and comparability. Therefore, it is essential to move beyond viewing tag‐induced effects as experimental anomalies and instead systematically integrate them as fundamental design variables in aggregation and LLPS research. To address this knowledge gap, the present review analyzes recent literature on the phase behavior and aggregation of IDPs and outlines three main objectives. First, we categorize the effects of tags, linkers, and labels on aggregation and LLPS into a framework of interconnected categories based on observed phenomena and predicted interaction changes. These categories are not mutually exclusive; rather, they represent co‐occurring dimensions of tag‐induced perturbations. Second, we examine case studies of representative proteins to provide concrete examples of how such structural modifications alter protein behavior. Third, we propose a strategic framework for experimental design, detailing minimal controls and reporting standards to account for tagging effects, thereby improving the reproducibility of future studies.

## 2 Mechanisms and Patterns of Tag‐Induced Effects

In the study of protein aggregation and LLPS, tags and chemical labels are widely used for purification, visualization, and detection. However, because these assembly processes are governed by the balance of weak multivalent interactions, including hydrophobic, aromatic, and electrostatic contacts, exogenous modifications frequently perturb the assembly equilibrium. Rather than acting as inert additions, tags can substantially alter nucleation kinetics, phase boundaries, maturation, and the types and morphologies of the resulting assemblies (e.g., amyloid fibrils, liquid droplets, or amorphous aggregates). The extent of perturbation is highly context‐dependent, varying with the target protein, attachment site, and linker composition. Consequently, tagged constructs can introduce unexpected artifacts that complicate data interpretation. To provide a framework for the subsequent case studies, this section summarizes the primary classes of tags and categorizes their common effects on protein assembly.

### 2.1 Primary Classes of Tags and Labels

#### 2.1.1 Affinity Tags

Affinity tags, including poly‐histidine (His), histidine affinity tag (HAT), glutathione S‐transferase (GST), maltose‐binding protein (MBP), Strep‐tag, and FLAG‐tag, are primarily utilized for protein purification and biochemical detection [18, 19, 20]. However, in some systems, these tags can modulate assembly behavior through direct self‐association or electrostatic rewiring [12, 21]. For instance, His‐ and HAT‐tags introduce histidine‐rich metal‐binding motifs that can alter local charge states and metal‐dependent interactions, whereas the GST‐tag possesses an inherent dimeric nature that can artificially promote the clustering of target proteins [22, 23, 24, 25, 26].

#### 2.1.2 Solubility and Folding Tags

MBP, GST, and small ubiquitin‐like modifier (SUMO) are widely used to enhance expression and improve the apparent solubility of aggregation‐prone proteins [27, 28]. Fluorescent tags such as sfGFP are also utilized for solubilization [29]. Although often cleaved before downstream assays, these tags can keep fusion constructs in a soluble, non‐assembling state until proteolytic removal, likely by suppressing self‐association and increasing the effective solubility of the construct [30, 31]. This artificial stabilization can lead to an overestimation of the intrinsic resistance to assembly of the target protein, making the timing of enzymatic cleavage and the influence of residual sequences critical for data interpretation.

#### 2.1.3 Fluorescent Proteins

Fluorescent proteins (FPs), such as green fluorescent protein (GFP) and red fluorescent protein (RFP) derivatives, are widely used to monitor spatiotemporal dynamics and droplet fusion in living cells [2], and GFP has also been used in some systems to improve the soluble expression of fusion proteins [29]. Because of their relatively large size (approximately 27 kDa), FPs can impose steric constraints and, depending on the variant, introduce residual self‐association or other non‐native surface properties [32]. These effects can alter not only the initial formation of droplets but also their number, size, and long‐term material properties, including fluidity and maturation into solid‐like states [33].

#### 2.1.4 Chemical Labels

Chemical labels include small covalent modifications such as fluorescent dyes, spin labels, and related probes used for visualization, tracking, or biophysical measurements. Unlike fusion tags, these labels add little steric bulk to the overall construct, but their effects can still vary with the labeling chemistry, attachment site, and degree of labeling [34]. Accurate comparisons across preparations, therefore, require a clear distinction between site‐specific and stochastic labeling strategies. Even small labels should be treated as experimental variables rather than neutral additions [35, 36].

### 2.2 Patterns of Tag‐Induced Effects

**Figure 2:**
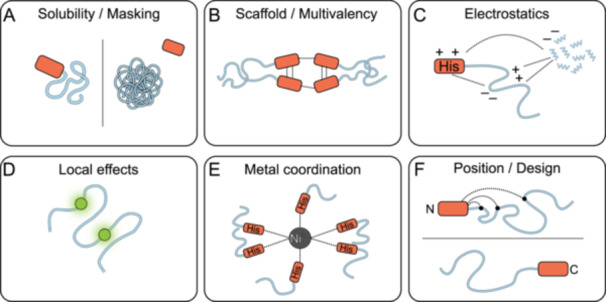
Six recurrent patterns of tag‐induced effects. Schematic overview of the six tag‐effect patterns defined in the main text. These patterns are non‐exclusive and can operate simultaneously within a single tagged construct. Fusion tags can perturb intermolecular interactions, phase behavior, and assembly pathways through multiple mechanisms. (A) Solubility and masking (Type I): large tags sterically occlude interaction‐prone regions and/or increase apparent solubility, thereby suppressing intrinsic assembly. (B) Scaffolding and multivalency (Type II): self‐associating tags create local high‐concentration hubs and increase effective valency, thereby promoting intermolecular interactions. (C) Electrostatic effects (Type III): charged tags, linkers, or cleavage scars alter electrostatic interactions and shift phase behavior. (D) Local effects on assembly pathways (Type IV): minor local modifications perturb specific sites and redirect competing assembly pathways. (E) Metal‐mediated coordination (Type V): His‐tags can coordinate transition metals, generating artificial cross‐links and conditional assembly states. (F) Positional and fusion‐design effects (Type VI): tag placement and fusion design, including terminal position and linker architecture, influence access to interaction‐prone regions and modulate the manifestation of other tag‐induced effects.

To facilitate the interpretation of diverse experimental observations, we group tag‐induced effects into six recurrent patterns (Figure 2). These patterns represent analytical categories rather than mutually exclusive molecular mechanisms. A single tag can simultaneously alter protein solubility, introduce new interaction surfaces, modify local charge distributions, perturb site‐specific contacts, and create conditional interactions through metal binding. Thus, multiple effects frequently coexist within a single construct, with the dominant pattern depending on the fusion design, solution conditions, and experiment type.

#### 2.2.1 Solubility and Masking (Figure 2A)

Large, globular tags can suppress phase behavior by steric occlusion and/or by increasing the apparent solubility of fusion constructs. Common examples include MBP, SUMO, GST, and FP fusions, which often exhibit reduced LLPS, delayed fibrillization, prolonged lag times, or a requirement for site‐specific proteolytic cleavage before intrinsic assembly becomes apparent. This property is often used to express and purify highly aggregation‐prone proteins before in vitro assembly assays [37].

#### 2.2.2 Scaffolding and Multivalency (Figure 2B)

Tags with self‐associating tendencies or stable dimeric structures, such as FPs and GST, can increase effective valency and local concentration [13]. The same physical principle is also used in optogenetic systems, in which light‐responsive modules promote assembly by increasing oligomerization and effective multivalency [38]. Depending on the protein and assay context, this can enhance nucleation and LLPS, ultimately shifting the system toward alternative assembly states. Consequently, the balance among liquid‐like condensates, amorphous aggregates, and ordered fibrils may change in a system‐dependent manner.

#### 2.2.3 Electrostatic Effects (Figure 2C)

Charged residues introduced through tags such as His‐tag, linkers, or cleavage scars can alter intra‐ and intermolecular electrostatic interactions. Altered electrostatics may affect salt sensitivity, shift phase boundaries, or modulate co‐condensation with polyanionic partners such as RNA. The direction and magnitude of these effects depend not only on net charge but also on charge patterning and on the position of the tag relative to interaction‐prone regions [13, 39].

#### 2.2.4 Local Effects on Assembly Pathways (Figure 2D)

A local covalent modification by a chemical label can alter the assembly pathway even when the overall increase in molecular weight is minimal. Such effects arise when a label perturbs a specific interaction‐prone region through hydrophobic, aromatic, or electrostatic changes, thereby shifting the balance among competing oligomerization and maturation pathways [34, 40]. The labeled protein may still assemble, but the distribution of intermediates, the dominant assembly pathway, and the final aggregate morphology may change. The magnitude of perturbations is often site‐dependent and varies with the degree of labeling [36].

#### 2.2.5 Metal‐Mediated Coordination (Figure 2E)

For constructs containing His‐tags, protein assembly frequently becomes sensitive to transition metals or residual purification‐derived metal ions. Metal coordination can promote intermolecular bridging or enrich specific assembly states, making phase behavior or oligomer distributions dependent on metal species, concentration, and chelating conditions. Consequently, metal‐induced bridging should be treated primarily as a conditional source of artifact rather than as a general consequence of His‐tagging [25].

#### 2.2.6 Positional and Fusion‐Design Effects (Figure 2F)

Rather than acting as an independent molecular mechanism, fusion design acts as a modifier that shapes the manifestation of the aforementioned effects. The effect of tagging is often influenced by fusion design, including N‐terminal versus C‐terminal placement, linker length, and residual amino‐acid scars after cleavage. These features can alter access to amyloidogenic motifs or intrinsically disordered prion‐like regions, helping explain why the same tag can produce different results across constructs. In this sense, positional and fusion‐design effects primarily shape how strongly the other effects become manifest in a given construct [13, 41].

## 3 Case Studies

Tag‐induced effects are rarely uniform across all experimental parameters. A tag may alter assembly kinetics while preserving the final structure, or it may change material properties, such as the liquid‐to‐solid transition, without affecting the initial formation of droplets. Therefore, summarizing tag effects simply as promoting or suppressing assembly is insufficient. Accurate interpretation requires distinguishing which specific property, such as saturation concentration, lag time, morphology, or fluidity, is perturbed. In the following case studies, we apply these mechanistic categories to clarify how specific tags influence the observed properties of various protein systems (Table 1). Because a single tag frequently induces multiple co‐occurring effects, we highlight the dominant tag‐effect types for each case, reflecting the primary layers of perturbation while acknowledging other potential contributions. The observed aggregation behavior is often the net result of competing or synergistic effects from multiple types. By deconstructing these observations into the six types, we can better rationalize why the same tag might yield different results depending on the cargo protein and experimental conditions.

**Table 1:** Representative Effects of Tagging on Protein Aggregation.

Category	Protein	Tag	Position/architecture	Affected property(ies)	Representative effect	Tag‐effect type(s)	Reference
Neurodegeneration	Aβ (1‐40)	HiLyte 488/647; Atto 488/655	N‐terminus; 5%/100% DOL	oligomer distribution; fibril morphology	Altered oligomer distribution and fibril morphology with preserved cross‐β core	IV	[35]
Neurodegeneration	Aβ (1‐42)	GFP	C‐terminus with short/long linker	assembly state	Stabilization of oligomeric states over mature fibrils; linker‐dependent	I/VI	[42]
Neurodegeneration	TDP‐43	His‐SUMO; MBP	solubility‐tag fusion; cleavage‐triggered assembly	solubility; assembly onset	Delayed assembly until tag cleavage	I	[30]; Wang et al. 2018
Neurodegeneration	Httex1 (Q25)	RFP	C‐terminal	LLPS; fibrillization; cell aggregates	Droplet formation and accelerated fibrillization under PEG, unlike untagged protein	II	[16]
RNP/condensates	CPEB3 (PRD1/PRD2)	His‐tag	N‐terminal	Assembly state; LLPS competence; fibril structure	PRD1: amorphous aggregates → fibrils; PRD2: droplets → amorphous aggregates	III	[12]
RNP/condensates	CPEB3 (full IDR)	His‐GFP	N‐terminal	LLPS vs amorphous aggregation	Full IDR: droplets → irregular amorphous aggregates	II/III	[12]
RNP/condensates	HP1α	mEGFP, AID‐sfGFP, UnaG, Atto488	C‐terminus; linker‐length tested	*C*_sat_; phase behavior; FRAP	Reduced LLPS with mEGFP; enhanced LLPS with AID‐sfGFP; linker dependence with UnaG; minimal perturbation with Atto488	I/III/VI	[43]
RNP/condensates	DDX3X	mCherry, mEGFP	C‐terminal	SG formation; condensate behavior	Reduced SG formation, with a milder effect for mEGFP	II/III/VI	[13]
RNP/condensates	DDX3X	myc/V5; Halo/SNAP	C‐terminal	SG morphology /recruitment	Near‐endogenous behavior with Myc/V5; smaller irregular SGs with Halo/SNAP; further impairment by dye labeling	I/III/VI	[13]
RNP/condensates	hnRNPA1	His‐SUMO	N‐terminal	salt dependence of phase separation	Reversed salt dependence of phase separation relative to the isolated hnRNPA1 LCD	III	[44]
Functional amyloids	FapB	His‐tag	N‐ vs C‐terminus	fibrillation competence	Fibril formation with N‐terminal tagging but not with C‐terminal tagging	VI	[45]

*Note:* “Tag‐Effect Type(s)” Indicates the Most Plausible Tagging‐Effect Category or Categories for Each Case.

Abbreviations: Csat, saturation concentration; DOL, degree of labeling; FRAP, fluorescence recovery after photobleaching; LCD, low‐complexity domain; LLPS, liquid–liquid phase separation; PEG, polyethylene glycol; PHF, paired helical filament; PRD, prion‐like domain; RNP, ribonucleoprotein; SG, stress granule.

### 3.1 Neurodegeneration and Pathogenic Amyloids

#### 3.1.1 Amyloid β

*Dominant Tag‐Effect Types: IV/VI; I in Cleavage‐Based Systems*

Amyloid β (Aβ) is a short peptide of 40 to 42 residues that serves as a central molecule in the pathogenesis of Alzheimer's disease and cerebral amyloid angiopathy [46, 47]. Aβ undergoes a nucleation‐dependent, multi‐step self‐assembly process from monomers to oligomers, protofibrils, and mature amyloid fibrils. This progression is highly sensitive to solution conditions [48]. To track the assembly process, small‐molecule fluorophores, such as AMCA, TAMRA, or HiLyte Fluor 488, are frequently conjugated to the N‐terminus of Aβ (1–40). Although the attachment of these dyes did not completely prevent protofibril or amyloid fibril formation as evaluated by transmission electron microscopy (TEM) [49], the specific fluorophore influences both the initial oligomer distribution and the final morphology. For example, the physicochemical properties of the N‐terminal dye altered the resulting oligomeric states; relatively hydrophilic dyes predominantly generated low‐molecular‐weight oligomers that were consumed during fibrillization, whereas dyes with higher hydrophobic interaction propensities stabilized high‐molecular‐weight oligomers that persisted in solution after 24 h [35]. These findings suggest that local changes in hydrophobicity, aromaticity, and charge near the N‐terminus can shift the initial oligomer distribution and subsequently influence the final fibril morphology. Atomic force microscopy (AFM) and TEM also revealed differences in fibril thickness and curvature depending on the attached dye [35]. Furthermore, X‐ray diffraction confirmed the preservation of the cross‐β reflection in dye‐conjugated assemblies, indicating that the core β‐sheet packing remains fundamentally unperturbed. However, given the 5% degree of labeling used in the study, such findings cannot be directly generalized to fully labeled preparations. Thus, the primary interpretational challenge with small fluorescent dyes in Aβ research is not whether fibrillization occurs, but how the specific label shifts the oligomerization pathway and the final morphological state.

The addition of large fluorescent proteins, such as GFP, requires cautious interpretation. In an *Escherichia coli* screening system based on Aβ (1–42)‐GFP fusions, highly aggregation‐prone Aβ sequences caused a loss of GFP fluorescence, whereas variants with low aggregation propensities retained fluorescence [50]. Although this fusion system serves as a useful screening tool, the presence or absence of fluorescence cannot be directly equated with the native assembly state of Aβ. For instance, Aβ (1–42)‐GFP fusions with optimized linker lengths allowed the visualization of oligomers both in vitro and in cells while maintaining fluorescence, but these fusion constructs formed stable oligomers rather than mature fibrils [42]. This stabilization may arise from the large steric bulk of GFP and the linker‐dependent restriction of Aβ–Aβ packing. Yeast models expressing GFP‐Aβ (1–42) or Aβ (1–42)‐GFP fusions also showed intracellular accumulation accompanied by fluorescence [51]. These observations suggest that GFP does not act merely as a passive visualization tag for Aβ. Instead, the fluorescent protein functions as an active structural determinant that selectively stabilizes specific assembly states and prevents the transition into canonical fibrils.

For in vitro production, the high aggregation propensity of Aβ often necessitates the use of solubility‐enhancing fusion tags, such as MBP or SUMO tags, followed by enzymatic cleavage to release the native Aβ sequence [37, 52]. In such experimental designs, a critical factor is the rate at which Aβ is released from the fusion construct, in addition to any effects from the residual tag sequence. The cleavage rate directly influences the kinetics of self‐assembly. During the cleavage of SUMO‐Aβ (1–40) by Ulp1, decreasing the enzyme‐to‐peptide ratio from 1:100 to 1:1000 increased the lag time 3.3‐fold, whereas the elongation rate remained largely unchanged [53]. In cleavage‐induced aggregation assays, the cleavage conditions should therefore be treated not as a simple preparatory step, but as an integral experimental variable that dictates the overall self‐assembly kinetics observed in subsequent measurements, such as Thioflavin T (ThT) fluorescence assays.

The effects of smaller affinity tags or minor sequence modifications are more subtle but can emerge under specific conditions. Recombinant Aβ (1–42) with a C‐terminal His‐tag retains its amyloidogenicity and cytotoxicity, and molecular dynamics simulations suggest that the His‐tag addition causes minimal structural perturbation [54]. However, Aβ fibrillization is highly pH‐dependent, and the midpoint of the pH dependence for primary nucleation closely aligns with the p*K*a of the native Aβ histidine residues [48]. Because His‐tags introduce additional ionizable groups in the critical pH range, construct‐dependent effects of the His‐tag may become apparent under specific buffer conditions. In contrast, the N‐terminal methionine residue, which often remains after recombinant expression, has a minimal impact. Met‐Aβ (1–42) and native Aβ (1–42) exhibit nearly identical aggregation kinetics, assembly mechanisms, and fibril core structures [55]. As a related consideration, although ThT is an extrinsic probe rather than a covalent tag, its physical presence can accelerate Aβ (1–40) fibril formation [56]. These studies indicate that the effects of tags and labels on Aβ should not be evaluated as a binary question of whether aggregation is inhibited or promoted.

#### 3.1.2 TDP‐43

*Dominant Tag‐Effect Types: I and VI*

TAR DNA‐binding protein 43 (TDP‐43) is an RNA‐binding protein consisting of an N‐terminal domain, two RNA recognition motifs, and a C‐terminal low‐complexity or prion‐like domain (PrLD). It is the primary component of pathological inclusions in amyotrophic lateral sclerosis (ALS) and frontotemporal dementia [57]. Because full‐length TDP‐43 exhibits an intrinsic propensity for self‐association, maintaining the protein in a soluble state in vitro is difficult. Consequently, solubility‐enhancing tags, such as MBP or SUMO, are often used for its expression and purification. In these expression systems, the bulky tag maintains solubility by suppressing self‐association. Subsequent proteolytic removal of the tag using a specific protease, such as TEV or Ulp1, triggers TDP‐43 assembly [30, 58].

While this cleavage‐induced approach is practically useful, the resulting observations should be interpreted within the context of the construct design. Analyses of full‐length TDP‐43 without large solubility tags showed that even when the protein formed spherical, droplet‐like structures, the internal diffusion of these assemblies remained restricted, indicating that they do not constitute simple liquid droplets [59]. In cleavage‐based assays, the bulky tag temporarily masks the strong intrinsic assembly propensity. Consequently, the true material properties of the protein become apparent only after the tag is removed [30, 58, 59]. Furthermore, comparisons between full‐length TDP‐43 and specific fragments demonstrated that the material properties of the assemblies formed by the isolated PrLD differed from those of the full‐length protein [60]. These differences confirm that sequences outside the PrLD significantly influence the final assembled state. Overall, although solubility tags allow for the preparation of TDP‐43 samples, the use of these tags and the specific timing of their cleavage directly dictate the onset of assembly and influence the interpretation of the resulting physical states.

#### 3.1.3 Httex1

*Dominant Tag‐Effect Type: II*

Huntingtin exon‐1 (Httex1) is the N‐terminal fragment of huntingtin, consisting of the N17 sequence, a polyglutamine (polyQ) tract, and a proline‐rich domain [61]. Pathogenic expansion of the polyQ tract drives oligomerization, amyloid fibril formation, and intracellular inclusions, making Httex1 a widespread model for studying Huntington's disease. Fusions with fluorescent proteins, such as GFP and RFP tags, are frequently used to visualize early aggregation processes and intermediate states. However, given the short and highly aggregation‐prone nature of Httex1, the addition of bulky fluorescent tags can significantly alter its self‐assembly equilibrium. For instance, a model proposing that Httex1 aggregation involves a liquid‐like intermediate state was based on the observation that GFP‐fused Httex1 formed droplet‐like assemblies before transitioning into solid fibrillar aggregates [62]. Because the principal evidence was derived from GFP‐fusion constructs in cells and in vitro assays, generalizing this liquid‐to‐solid transition to the intrinsic behavior of native, untagged Httex1 requires careful consideration.

Directly addressing the construct‐dependent difference, a recent study compared Httex1 (Q25) fused to a C‐terminal RFP with untagged Httex1 (Q25) [16]. Under macromolecular crowding conditions, RFP‐tagged Httex1 (Q25) rapidly formed micron‐sized phase‐separated droplets that subsequently progressed to gelation and fibrillization. In contrast, untagged Httex1 (Q25) did not form detectable liquid condensates under identical conditions. Furthermore, in cellular contexts, RFP‐tagged Httex1 formed aggregates that differed in size and staining properties from those formed by the untagged protein. These structural and morphological deviations indicate that the RFP tag does not function merely as a passive visualization marker but actively redirects the aggregation pathway and assembly progression of Httex1. Although speculative, weak self‐association between RFP moieties might contribute to this RFP‐dependent phase separation.

Interpretational complexities also arise in systems utilizing solubility tags. An intein‐based strategy used to generate native, untagged Httex1 revealed aggregation behaviors distinct from those observed in fusion‐based or *in situ* cleavage systems [63]. The strictly untagged system demonstrated that even Httex1 variants with non‐pathogenic polyQ lengths formed fibrils, an assembly propensity not consistently observed in cleavage‐based assays. Because the simple co‐incubation of GST or MBP with untagged Httex1 did not significantly alter its aggregation properties, the behavioral discrepancies in fusion‐based systems likely stem from a combination of the physical tethering of the tag, incomplete proteolytic cleavage, and the resulting sample heterogeneity.

Collectively, these findings indicate that tag effects in Httex1 are not confined to variations in detection signals. Instead, tags actively dictate which assembly states are favored and how the aggregation process unfolds. Therefore, it is crucial to validate mechanistic models of Httex1 assembly using untagged preparations or constructs with minimal residual scars to ensure that the observed phenotypes reflect the intrinsic properties of the protein rather than design‐dependent differences.

#### 3.1.4 SOD1, PrP

*Dominant Tag‐Effect Types: VI; I in Cleavage‐Based PrP Systems*

Superoxide dismutase 1 (SOD1) is an antioxidant enzyme frequently mutated in familial ALS [64], whereas the prion protein (PrP) causes prion diseases through a structural conversion from a cellular isoform into an aggregated state [65]. In both protein systems, differences in tag addition or construct design can substantially alter the observed assembly states and functional evaluations.

For SOD1, the presence of a tag can directly dictate the final assembly state. Specifically, untagged wild‐type SOD1 and the G93A variant formed amyloid‐like fibrils under specific in vitro conditions, whereas GFP‐fused SOD1 G93A failed to display even initial oligomerization under identical conditions [66]. This morphological divergence indicates that for SOD1, GFP does not act simply as an observational marker but rather shifts the entire self‐assembly pathway.

Conversely, the effects of a His‐tag on PrP assembly manifest primarily as kinetic and structural shifts rather than an absolute block of fibrillization. Truncated human prion protein (HuPrP) constructs with an N‐terminal His‐tag, such as His‐HuPrP (90–231) and His‐HuPrP (121–231), formed amyloid‐like fibrils similarly to their untagged counterparts [67]. However, fibrillization kinetics varied among these constructs. His‐HuPrP (121–231) and HuPrP (121–231) exhibited the shortest lag times (0.6 ± 0.2 h and 0.9 ± 0.3 h, respectively), followed by HuPrP (90–231) (1.2 ± 0.2 h), while His‐HuPrP(90–231) showed the longest (2.3 ± 0.4 h). Furthermore, untagged constructs yielded higher average ThT fluorescence intensities at equilibrium. These kinetic differences suggest that for certain truncated PrP constructs, the His‐tag alters the fibrillization kinetics and the specific fibrillar conformations detected by ThT, rather than determining the fundamental ability to form fibrils.

Beyond fibrillization, studies of PrP LLPS frequently employ MBP or GFP fusion constructs, where condensation is triggered by TEV protease cleavage [68]. In the cleavage‐induced systems, the construct design itself governs the emergence of phase behavior and influences the resulting measurements. Furthermore, given the critical role of the PrP N‐terminal intrinsically disordered region (IDR), introducing tags or modifications near the N‐terminus can confound the results of domain‐specific functional assays [69].

Therefore, rather than simply categorizing tags as generic promoters or inhibitors of aggregation, these cases illustrate that specific tags modulate the final assembled state, assembly kinetics, and structural features captured by standard measurement techniques.

#### 3.1.5 Tau, FUS, α‐Synuclein

*Dominant Tag‐Effect Types: I, IV and VI*

Tau [70], fused in sarcoma (FUS) [71], and α‐synuclein [4] are closely associated with neurodegenerative diseases and exhibit multiple self‐assembly states, including oligomers, amyloid fibrils, and biomolecular condensates. Given their structural versatility, the effects of tags and small‐molecule labels manifest differently across various assembly stages and experimental measurements. For example, in Tau, which is primarily linked to Alzheimer's disease and related tauopathies, the impact of a GFP tag depends heavily on the specific protein domain evaluated [15]. Fusing GFP to the isolated repeat domain of Tau strongly inhibited the formation of amyloid fibrils with the characteristic paired helical filament (PHF) morphology, yielding assemblies that did not accurately represent Alzheimer‐type Tau fibrils. Conversely, GFP‐fused full‐length Tau retained the capacity to form fibrils, but light‐scattering assays showed that the tag altered the apparent aggregation kinetics and final scattering output. Untagged full‐length Tau (TauFL‐wt) aggregated mainly into PHF‐like filaments with a half‐time of approximately 3 h, whereas GFP‐TauFL‐wt and TauFL‐wt‐GFP aggregated with half‐times of approximately 2 h and 12 h, respectively. Both GFP‐fused constructs also reached higher final light‐scattering levels, consistent with their increased subunit mass and altered elongation behavior. These domain‐dependent differences indicate that GFP imposes a substantial steric hindrance, particularly within or near the repeat‐domain assembly core, rather than universally inhibiting fibrillization.

For FUS, a protein implicated in ALS and frontotemporal dementia (FTD), the influence of fluorescent tags varies depending on both the specific tag type and the experimental conditions. Comparative studies demonstrated that an mCherry tag significantly reduced condensate formation, whereas an mEGFP tag exerted a relatively minor effect on the phase boundary [13]. Beyond fluorescent labels, in vitro studies of FUS frequently employ a construct design in which an MBP fusion is proteolytically cleaved to initiate assembly. This cleavage step is essential for observing the intrinsic phase behavior of FUS, demonstrating that the presence and subsequent removal of the tag directly dictate the onset of condensation [72].

In the case of α‐synuclein, the hallmark protein of Parkinson's disease and Lewy body dementias, the introduction of tags or chemical labels frequently alters the specific fibril morphology and the intensity of detection signals, rather than completely abolishing the capacity for fibril formation. In A140C α‐synuclein labeled with ATTO 655 or Alexa 647, increasing the fraction of labeled protein shortened the fibrils while leaving their height largely unchanged. Notably, 100% labeling abolished the characteristic twisted morphology observed in wild‐type and unlabeled A140C fibrils. Because dyes with different net charges produced similar morphological changes, this effect was attributed mainly to local steric or structural perturbation by closely spaced dye molecules rather than to simple electrostatic effects [36]. Similarly, our recent comparison between His‐tagged and untagged α‐synuclein showed that the His‐tag suppressed the maximum fluorescence intensity of ThT, even though TEM confirmed that the His‐tagged α‐synuclein successfully formed canonical amyloid fibrils [12].

Collectively, these examples demonstrate that within proteins capable of multiple assembly states, the presence of a tag or chemical label yields diverse phenotypes. These construct‐dependent effects span from altered fibril morphology and shifted phase separation thresholds to differences in fluorescent probe binding. Consequently, interpreting self‐assembly properties for these proteins requires a comprehensive evaluation that accounts for the specific tag type, the attachment site, the construct design, and the analytical techniques utilized.

#### 3.1.6 Ataxin‐2, C9orf72 dipeptides, IAPP, TTR

*Dominant Tag‐Effect Types: Unassigned (Unresolved Effects)*

Ataxin‐2, *C9orf72* dipeptide repeat (DPR) proteins, islet amyloid polypeptide (IAPP), and transthyretin (TTR) constitute a group of proteins in which the direct effects of tags on intrinsic aggregation are either minimal or difficult to clearly define, making it challenging to isolate the exact stage of assembly perturbed by these modifications.

For Ataxin‐2, a protein involved in RNA metabolism and SG formation, the LCDs of both Ataxin‐2 and its yeast ortholog Pbp1 form condensates [73]. However, most in vitro studies utilized His‐tagged constructs, whereas in vivo experiments relied on fluorescent or SNAP tags [74, 75, 76]. Because direct comparisons between these tagged constructs and untagged Ataxin‐2 or Pbp1 are limited, evaluating the independent contribution of these tags to LLPS or self‐assembly remains difficult.

In the case of *C9orf72* DPRs, which are short aberrant peptides generated via repeat‐associated non‐AUG translation from expanded *C9orf72* alleles in ALS and FTD [77], tags predominantly alter downstream cellular phenotypes rather than strictly defining the initial assembly. For instance, in *Drosophila* models, a C‐terminal mCherry tag exacerbated the toxicity of short arginine‐rich DPRs. Conversely, the addition of a GFP, mCherry, or FLAG tag significantly reduced the toxicity of polyGA peptides [17]. Although these modifications clearly influenced toxicity, stability, and cellular responses, it is unclear whether these phenotypic shifts stemmed from alterations in the intrinsic aggregation propensity of the DPRs or from differences in post‐aggregation localization, degradation, and cellular interactions.

For IAPP, a 37‐residue peptide hormone that forms amyloid deposits in type 2 diabetes, while the standard model for aggregation assays relies on untagged synthetic peptides [78], cryo‐electron microscopy (cryo‐EM) studies using recombinant full‐length human IAPP fused to an N‐terminal SUMO tag demonstrated that the tagged protein still formed amyloid fibrils. The ordered core corresponded to residues 14–37, with no resolved density for the SUMO tag, indicating that the SUMO addition did not sterically prevent fibril core formation [79]. Despite the structural compatibility in vitro, in vivo models present interpretational challenges. In *Caenorhabditis elegans* models, IAPP‐GFP fusion proteins formed solid aggregates with a filamentous appearance under TEM. Yet, they were neither detected by the amyloid‐binding dye NIAD‐4 nor caused severe motility defects [80]. This discrepancy indicates that assemblies visualized in tagged samples cannot be automatically equated with native, pathogenic amyloid structures.

Finally, TTR, a tetrameric transport protein that causes ATTR amyloidosis upon dissociation, is relatively tolerant to certain tags, although exceptions exist [81]. For example, a His‐tag did not alter the aggregation behavior of TTR during acid‐mediated aggregation assays [82]. In contrast, native ion mobility‐mass spectrometry revealed that an N‐terminal dual‐FLAG tag altered the quaternary structure and thermodynamic stability of the tetramer [83]. Therefore, even in systems where aggregation proceeds similarly, small affinity tags can subtly perturb structural stability.

### 3.2 RNP (RNP Granules/Phase Separation–Prone RBPs)

#### 3.2.1 CPEB3

*Dominant Tag‐Effect Types: II/III*

Cytoplasmic polyadenylation element‐binding protein 3 (CPEB3) is an RNA‐binding protein critical for the maintenance of long‐term memory [84, 85]. It contains an N‐terminal intrinsically disordered region (IDR), which exhibits both liquid droplet and amyloid formation in vitro. Within cellular environments, its localization to P‐bodies and its role in translational regulation have been characterized in detail [86]. To facilitate purification and visualization, studies of CPEB3 routinely employ fusions with His‐tags, GFP, or DsRed.

Our recent comparative analysis using mouse CPEB3 fragments revealed that the IDR comprises two distinct regions with unique assembly profiles: the amyloid‐prone prion domain 1 (PRD1) and the droplet‐forming prion domain 2 (PRD2) [12]. We demonstrated that fusion tags do not merely alter the kinetics of assembly but induce a qualitative departure from the native assembly pathways of these specific domains. Specifically, the addition of a His‐tag to PRD1 promoted the formation of amyloid fibrils within a domain that otherwise forms amorphous aggregates in its untagged state. The resulting fibrils exhibited an α‐helix‐rich secondary structure, deviating from the structural hallmarks of other CPEB family proteins. Conversely, the His‐tag altered the temperature‐dependent turbidity profile of PRD2, the domain driving LLPS. Whereas untagged PRD2 showed reduced turbidity at 95°C, His‐tagged PRD2 remained highly turbid. Although a modest turbidity increase upon cooling indicated some residual droplet‐like behavior, substantial amorphous aggregation also occurred. These findings suggest that the additional histidine residues from the tag perturb the balance of electrostatic interactions.

These findings indicate that the assembly states, such as droplets or fibrils, observed in tagged constructs may not faithfully reflect the molecular properties of native CPEB3. The structural alterations induced by tags represent a fundamental shift in the self‐assembly landscape of PRD1 and PRD2. This shift highlights the necessity of validating observations using untagged or minimally modified systems to ensure the biological relevance of the observed protein assemblies.

#### 3.2.2 HP1α

*Dominant Tag‐Effect Types: I/III/VI*

Heterochromatin protein 1α (HP1α) is a central regulator of heterochromatin formation and maintenance [87]. Its capacity to undergo LLPS, driven by DNA binding or N‐terminal phosphorylation, has made it a foundational model for studying biomolecular condensation in the context of gene silencing. HP1α represents one of the few protein systems where tag‐induced perturbations have been systematically evaluated across multiple experimental modalities.

A recent comprehensive comparison of C‐terminally tagged HP1α constructs revealed that the specific choice of tag can qualitatively alter the resulting phase behavior [43]. The evaluated modifications included mEGFP, AID‐sfGFP, UnaG, and small‐molecule Atto488 labeling via a KCK‐linker. In the presence of 30 nM 2.7 kbp DNA, untagged HP1α and HP1α‐KCK‐Atto488 phase separated at a *C*_sat_ of 3.3 μM, whereas HP1α‐16aa‐mEGFP remained soluble up to 450 μM and HP1α‐8aa‐mEGFP formed non‐spherical phases only at 250 μM. These results show that the mEGFP tag strongly suppresses HP1α LLPS even under DNA‐containing conditions. In contrast, AID‐sfGFP exerted the opposite effect, lowering the *C*_sat_ to 1.3 μM and actively promoting condensation. While the smaller fluorescent protein UnaG was less perturbative than mEGFP, this tag effect depended on linker length and cofactor state: HP1α‐16aa‐holo‐UnaG phase separated at approximately 6.5–6.9 μM, whereas HP1α‐8aa‐holo‐UnaG did not phase separate up to 30 μM. Finally, Atto488 labeling via the KCK linker caused little detectable shift in *C*_sat_ under the tested conditions.

These findings indicate that tag effects are not merely a function of molecular weight or size. Instead, the specific physicochemical properties of the tag and the flexibility afforded by the linker architecture dictate the assembly threshold. Furthermore, environmental factors significantly modulate phase behavior. The addition of 10% polyethylene glycol (PEG)‐8000, a common macromolecular crowding agent, promoted LLPS across all constructs to an extent that masked the inherent behavioral differences between tags. Consequently, relying on data obtained under high‐crowding conditions may provide a misleading sense of construct neutrality. Collectively, the case of HP1α demonstrates the necessity of verifying phase behavior using a combination of different tags, linker lengths, and environmental contexts to distinguish intrinsic protein properties from design‐dependent artifacts.

#### 3.2.3 DDX3X, Pab1, Dhh1, EDC3

*Dominant Tag‐Effect Types: I/II/III*

DDX3X [88], Pab1 [89], Dhh1 [90], and EDC3 [91] function in RNA metabolism and regulate the formation of ribonucleoprotein (RNP) granules, such as SGs and P‐bodies. Within these multi‐component systems, the effects of fluorescent and affinity tags extend beyond the simple presence or absence of condensation. Instead, these modifications alter specific granule properties, including granule number, size, morphology, internal dynamics, and the co‐partitioning of interacting proteins.

The choice of fluorescent tags influences the condensation and granule‐partitioning behaviors of multiple RNA‐binding proteins. For example, the tag on the DEAD‐box RNA helicase DDX3X altered the morphology and enrichment of cellular SGs and modified the fluorescence recovery after photobleaching (FRAP) behavior of in vitro condensates; the FRAP half‐time was approximately 100 s for untagged DDX3X visualized with ATTO488 spike‐in, approximately 50–70 s for EGFP‐, mEGFP‐, or EYFP‐tagged DDX3X, and approximately 25 s for mCherry‐tagged DDX3X [13]. Similarly, the poly(A)‐binding protein Pab1 showed tag‐dependent differences in SG formation in yeast: mCherry and mScarlet‐I nearly abolished SG formation, whereas EYFP increased SG number by approximately twofold. For the P‐body‐associated DEAD‐box ATPase Dhh1, tags specifically altered the condensation threshold and the permissive pH range for phase separation in vitro [92]. Whereas untagged Dhh1 formed condensates at approximately 1 µM, GFP‐Dhh1 and mCh2‐Dhh1 required approximately 2 and 5 µM, respectively. The permissive pH range was also narrowed from pH 5.8–7.4 for untagged Dhh1 to pH ≤ 6.8 for GFP‐Dhh1 and pH ≤ 6.0 for mCh2‐Dhh1. In contrast, the addition of an N‐terminal His‐tag partially counteracted this inhibition. Even in 2% spike‐in experiments, tag identity influenced the apparent FRAP recovery of Dhh1 within condensates. In vivo, the identity and position of fluorescent tags fused to Dhh1 changed the number of P‐bodies during glucose starvation and, for some constructs, promoted constitutive foci even under glucose‐rich conditions. For the P‐body scaffold EDC3, mCherry fusion reduced P‐body number and also decreased the recruitment of DCP1‐ and DDX6‐positive foci. These observations indicate that tags can actively modulate the assembly behaviors of fusion constructs, rather than acting solely as passive reporters.

Collectively, these observations indicate that tags in RNP systems perturb not only the self‐assembly of the target molecule but also the composition and physical properties of the entire granule ecosystem. Mechanistically, these effects are most consistent with tag‐dependent changes in electrostatic balance and weak multivalent interactions, with tag position further shaping how these effects appear in cells. Consequently, phenotypic outputs from tagged constructs should not be directly equated with native granule behavior.

#### 3.2.4 hnRNPA1, Nab2

*Dominant Tag‐Effect Type: I/II/III*

Tags can alter the physicochemical requirements for phase separation, such as salt sensitivity and RNA dependence. For instance, hnRNPA1, an RNA‐binding protein, localizes to SGs and undergoes phase separation driven by its LCD [93]. In this system, a folded solubility tag shifted the salt‐dependent phase boundary. The isolated tag‐cleaved LCD showed stronger phase separation at elevated NaCl concentrations, whereas His‐SUMO‐tagged LCD showed the opposite trend, resembling the behavior of tag‐cleaved hnRNPA1 [44]. The suppressive effect under high‐salt conditions was especially evident for His‐SUMO–hnRNPA1, which failed to phase separate above 250 mM NaCl even at protein concentrations up to approximately 1200 µM. It has been proposed that folded domains and solubility tags change the interaction balance of the LCD: at low ionic strength, electrostatic interactions between the folded domain or tag and the LCD can support condensation, whereas at higher ionic strength these interactions are screened and the folded domain or tag instead increases the apparent solubility of the construct. A similar alteration in assembly requirements was observed for Nab2, a yeast nuclear poly(A)‐binding protein that natively forms RNA‐dependent condensates [94]. A Nab2‐ymEGFP construct retaining a His‐TwinStrep tag formed condensates independent of RNA specificity, assembling with non‐specific poly(U) RNA or even in the complete absence of RNA. In contrast, removal of the tag restored the strict RNA dependence of condensate formation [13]. These observations demonstrate that appended tags can alter not only the overall propensity for self‐assembly but also the specific conditions required for condensate formation, including salt sensitivity and RNA specificity.

#### 3.2.5 G3BP1, SRRM2, DDX6

*Dominant Tag‐Effect Types: Unassigned (Unresolved Effects)*

G3BP1 [95], DDX6 [96], and SRRM2 [97] are well‐established components of stress granules (SGs), P‐bodies, and nuclear speckles, respectively. G3BP1 functions as a central node that drives stress granule assembly, DDX6 is required for P‐body assembly, and SRRM2 is a core scaffold essential for nuclear speckle formation. In contrast to the previously discussed RNA‐binding proteins, which exhibited altered condensation properties upon tagging, these three proteins displayed relatively low sensitivity to fusion tags. Appending various fluorescent or peptide tags to the C‐terminus of G3BP1 caused minimal changes to the number, size, and enrichment level of SG foci [13]. Similarly, C‐terminal tagging of DDX6 did not appreciably alter P‐body appearance. For SRRM2, variations in protein expression levels had a greater effect on nuclear speckle properties than the choice of fusion tag. These comparative examples indicate that tag sensitivity is not uniform across condensate‐forming proteins. The degree of tag‐induced perturbation depends on the intrinsic properties of the individual target protein, even among components that co‐localize within the same biomolecular condensate.

#### 3.2.6 TIA‐1, LAF‐1, Whi3, FIB1

*Dominant Tag‐Effect Types: I/III/V*

Tags can alter both the initiation kinetics and the subsequent material properties of condensates formed by RNA‐binding proteins and nuclear components, such as TIA‐1 [98], LAF‐1 [99], Whi3 [100], and FIB1 [101]. TIA‐1 promotes stress granule assembly, LAF‐1 is a P‐granule‐associated DEAD‐box RNA helicase, Whi3 binds the CLN3 mRNA to regulate cell fate and cell‐cycle entry, and FIB1 (fibrillarin) is a nucleolar dense fibrillar component (DFC) protein. For the prion‐related domain (PRD) of TIA‐1, fusion with GFP artificially enhanced protein solubility [102]. This increased solubility delayed the onset of microaggregation and consequently impaired SG assembly. A similar suppressive effect occurred in the isolated RGG domain of LAF‐1, where an MBP fusion maintained the target sequence in a highly soluble state. Whereas the cleaved RGG construct underwent phase separation at concentrations above approximately 1 µM in 150 mM NaCl at pH 7.5, 6 µM MBP‐RGG remained soluble prior to protease addition. Rapid droplet formation was initiated only upon proteolytic removal of the MBP tag, and the droplet diameter reached a plateau within approximately 2 h after cleavage [103]. These observations demonstrate that large functional tags can strongly inhibit intrinsic phase separation. In contrast, specific tag placements can actively drive condensation. For example, appending an N‐terminal His‐tag to Whi3 enabled its artificial recruitment to supported lipid bilayers in a Ni‐NTA lipid‐based reconstituted system, thereby locally promoting condensate formation [104]. This targeted recruitment illustrates how affinity tags can artificially dictate phase behavior. Finally, even when tags do not completely block condensation, they can alter the physical properties of the resulting droplets. For FIB1, GFP tagging preserved the ability to form droplets but modulated quantitative dynamics, as shown by the slower relaxation of fused droplets. Specifically, FIB1::GFP droplets exhibited an inverse capillary velocity of 40 ± 10 s/µm, whereas FIB1:monoGFP and untagged FIB1 showed lower values of 15 ± 2 and 16 ± 2 s/µm, respectively [3]. GFP tagging also shifted the phase boundary, allowing FIB1 droplets to form at higher salt concentrations than the untagged protein.

### 3.3 Viral Condensates and Inclusions

#### 3.3.1 μNS

*Dominant Tag‐Effect Types: II/VI*

The mammalian *orthoreovirus* protein μNS is a primary scaffold of viral factories and forms factory‐like inclusions when expressed alone [105]. μNS has been characterized as a phase‐separating protein that forms dynamic, liquid‐like condensates [33]. Although fusion tags, including GFP variants and HaloTag, are routinely used to visualize these condensates, the tags themselves can substantially alter the condensation process. A systematic comparison of untagged μNS with N‐terminally tagged constructs showed that fluorescent tags altered μNS condensation primarily by changing inclusion density. mEGFP and moxGFP produced the strongest effect, increasing μNS inclusion density by approximately 6.6‐fold relative to untagged μNS. FusionRed and Dendra2 produced more moderate effects, increasing inclusion density and partially restoring condensation of noncondensing μNS mutants. In contrast, mNeonGreen showed the least perturbation among the GFP‐like tags, with inclusion density closest to that of untagged μNS. HaloTag increased condensate size without comparably increasing condensate density. Appending a fluorescent tag to a phase‐separation‐deficient μNS mutant artificially restored condensate formation. This restoration indicates that fluorescent tags can generate false‐positive condensation phenotypes, possibly by introducing additional scaffolding interactions, including FP–FP or FP–μNS contacts, that increase the apparent self‐association propensity of the fusion construct.

Tag position also influences the assembly state. While N‐terminal tags generally promote μNS condensation, earlier work demonstrated that a C‐terminal GFP fusion partially interfered with μNS inclusion formation [105]. This partial interference is consistent with the requirement of the carboxyl‐proximal region for μNS assembly [106]. Furthermore, the structural sensitivity of μNS extends to minimal tags. The insertion of a six‐residue tetracysteine tag prevented recombinant virus rescue in a site‐dependent manner, indicating that even small tags disrupt native interaction mechanisms [107].

Collectively, these observations demonstrate that visualization tags in the μNS system act as active determinants of condensation. Consequently, μNS illustrates both false‐positive phase separation induced by artificial tag properties and positional interference with native assembly domains.

#### 3.3.2 Rabies Virus N/P, VP1, SARS‐CoV‐2 Nucleocapsid Protein (N)

*Dominant Tag‐Effect Types: I/II/III/VI*

Rabies virus N/P complexes form Negri body‐like viral factories that support viral transcription and replication [108], while the SARS‐CoV‐2 nucleocapsid (N) protein is an RNA‐binding genome‐packaging protein that forms condensates with viral RNA [109]. Additionally, the foot‐and‐mouth disease virus (FMDV) capsid protein VP1 has been used as an aggregation‐prone viral model in recombinant inclusion‐body systems [110]. For the rabies virus N/P complex, the position of the fluorescent tag strongly influenced assembly behavior. Specifically, an N‐terminal GFP fusion to N formed condensates with P, whereas GFP insertion into specific internal flexible‐loop positions impaired condensate formation; furthermore, a mutant with GFP inserted between residues 106 and 107 within the flexible L1 loop of N exhibited a dominant‐negative effect on infection [111]. FRAP analysis further showed that P remained mobile whereas GFP‐N did not, indicating that tag placement altered both condensate formation and internal dynamics [111]. In a distinct recombinant model, appending a C‐terminal His‐tag to VP1‐GFP increased the size of bacterial inclusion bodies and the cross‐β structure content relative to the untagged construct [112]. For SARS‐CoV‐2 N, an N‐terminal MBP fusion was used to maintain solubility, and LLPS occurred only after TEV‐mediated cleavage of the MBP tag, indicating that the solubility tag suppressed LLPS prior to its removal [113]. Collectively, these examples demonstrate that tag type, placement, and cleavage state substantially influence the assembly of viral or virus‐derived proteins in experimental systems.

### 3.4 Functional Amyloids

#### 3.4.1 CsgA, FapC/FapB

*Dominant Tag‐Effect Types: III/VI*

CsgA [114] and Fap [115] proteins are functional amyloids that form a structural scaffold in bacterial biofilms. Although in vitro analyses often use recombinant His‐tagged CsgA constructs [116], fibrillization has also been reported for untagged preparations [117]. However, direct comparisons between tagged and untagged constructs under identical conditions remained absent. This lack of comparative data obscures the specific contribution of the His‐tag to CsgA assembly kinetics.

In the *Pseudomonas* Fap system, FapC is the major fibril‐forming subunit, whereas FapB plays a minor nucleating role [118]. Within this system, the effects of His‐tag placement are more clearly defined [45]. Fibrillization kinetics of FapC showed minimal differences between N‐ and C‐terminally tagged constructs. Conversely, tag position dictated FapB assembly; the N‐terminally tagged variant formed fibrils with a lag time of approximately 10 h at pH 5, whereas the C‐terminally tagged construct showed almost no fibrillization over 60 h across pH 5 to 9. Structural modeling indicated that the C‐terminal tag on FapB interacted with the protein surface via a short linker, altering the local charge distribution and preventing fibril formation. Moreover, the proteolytic removal of the tag triggered rapid fibrillization, which complicates accurate kinetic measurements of untagged recombinant samples. Collectively, these findings demonstrate that simple affinity tags can alter functional amyloid fibrillization in a position‐dependent manner, as observed with FapB, likely through changes in local charge distribution and electrostatic interactions.

#### 3.4.2 VHL

*Dominant Tag‐Effect Type: I/II*

Von Hippel‐Lindau protein (VHL) is a stress‐responsive protein implicated in A‐body biogenesis [119]. In vitro, VHL formed physiological amyloid fibrils upon incubation at acidic pH [26]. A direct comparison revealed that under these conditions, an N‐terminal GST fusion altered both the assembly pathway and the final fibril morphology of the protein. Specifically, whereas untagged VHL formed short fibrils (up to 300 nm in length and 8 nm in height), GST‐VHL produced longer, less rigid fibrils with resolvable chirality and greater hierarchical organization, exhibiting lengths of 200 nm to 20.7 μm and heights of 1.2 to 35 nm. This morphological shift is consistent with a scaffolding effect; because GST is a stable dimeric tag, it may constrain some early assembly states and promote uniform fibril growth. Although early intermediates remain unresolved, the VHL system demonstrates how a purification tag changes multiple observed aggregation properties, modifying both the efficiency of assembly and the final structural state of a functional amyloid.

### 3.5 Yeast Prions/Prion‐Like Proteins

#### 3.5.1 Sup35

*Dominant Tag‐Effect Type: VI*

Sup35 is the yeast translation termination factor eRF3, which forms a heritable prion state known as [*PSI* ^+^] [120, 121]. This prion property relies on the N‐terminal prion‐forming region within the NM domain. In the Sup35 system, the structural and functional consequences of tagging are highly sensitive to the insertion site and overall construct design. For example, appending a His‐tag to the Sup35 NM fragment yielded position‐dependent effects [122]. An N‐terminally tagged construct formed amyloid fibrils structurally comparable to those of the untagged protein, whereas a C‐terminal His‐tag increased fibril polymorphism. This suggests that the C‐terminal His‐tag changes intra‐ or intermolecular contacts within Sup35 NM. Similarly, the impact of a GFP tag depends on its placement. When GFP was inserted internally between the N and M domains of full‐length Sup35, the aggregation pattern differed from that of terminal fusions. This internally tagged construct did not form visible fluorescent foci in log‐phase cells and formed foci only in aging cultures, yet it preserved translation termination activity and maintained the [*PSI* ^+^] state more effectively than terminal fusions [123]. Separately, the MBP tag is frequently utilized as a solubility tag to suppress early self‐assembly and maintain the protein in a soluble state prior to experimental triggers [124]. Collectively, these observations demonstrate that for Sup35, the specific placement and structural context of the tag govern the assembled state and physiological properties more strongly than the basic physicochemical identity of the tag.

#### 3.5.2 Ure2

*Dominant Tag‐Effect Type: VI*

The yeast nitrogen metabolism regulator Ure2 underlies the [*URE3*] prion phenotype through self‐assembly [125]. Initial GFP‐based overexpression systems showed that full‐length Ure2‐GFP and prion‐domain constructs, such as Ure2(1–65)‐GFP, aggregated specifically in [*URE3*] cells, and overexpression of Ure2‐GFP fusion proteins cured [*URE3*] [126]. Under these conditions, Ure2‐GFP formed numerous large fluorescent foci during prion elimination, and these large aggregates were interpreted as dead‐end products rather than transmissible propagons, whereas the transmissible propagons remained soluble [127]. To reduce this perturbation, GFP was inserted internally after residue 90 of full‐length Ure2, rather than fused at the C terminus or inserted after residue 65 [41]. When this internally tagged construct was expressed at relatively low levels, neither the mitotic stability of [*URE3*] nor its curing rate was detectably altered. These results indicate that the effect of GFP on Ure2 assemblies depends not only on the presence of the tag, but also on its fusion position and the overall expression level.

### 3.6 Membrane Proteins

#### 3.6.1 Adic, Proteorhodopsin

*Dominant Tag‐Effect Types: II/III*

The effect of protein tagging on membrane proteins often manifests as the artificial emergence of non‐native higher‐order assemblies. The arginine/agmatine antiporter AdiC [128] and the light‐driven proton pump proteorhodopsin [129] exemplify this phenomenon, as both proteins adopt specific oligomeric states within the membrane. In their untagged forms, AdiC exists as a dimer, and proteorhodopsin forms a pentamer [130]. However, the addition of a His‐tag disrupted the native states, inducing AdiC to form tetramers and proteorhodopsin to assemble into decamers and pentadecamers. Size‐exclusion chromatography and single‐particle cryo‐EM confirmed that the higher‐order oligomers were driven directly by interactions between the His‐tags rather than intrinsic protein interfaces [21]. Therefore, these observations demonstrate that even short affinity tags can artificially alter the native oligomeric state of membrane proteins.

### 3.7 Miscellaneous Systems

#### 3.7.1 Androgen Receptor (AR) NTD

*Dominant Tag‐Effect Types: I/II*

The N‐terminal domain (NTD) of the androgen receptor (AR) is an IDR of approximately 560 residues that contains a polymorphic polyQ tract [131]. Studies examining the self‐association and amyloid‐like assembly of the AR‐NTD have compared tagged and untagged constructs [132]. Specifically, the interplay between the KELCKAVSVSM motif and the polyQ tract was investigated using His‐SUMO‐fused peptides, GST‐tagged fragments, and untagged variants. In the His‐SUMO fusion system, the KELCKAVSVSM peptide remained primarily as monomers or covalent dimers without forming fibrils; however, subsequent SUMO cleavage and exposure of the purified peptide to DMSO‐containing conditions triggered its assembly into fibrils. Furthermore, AFM revealed that although GST‐tagged AR‐NTD fragments and their untagged counterparts yielded broadly similar oligomeric species, the fibrillar oligomers derived from the GST‐tagged fragments were thicker. These observations indicate that fusion tags can alter the morphology and apparent assembly behavior of AR‐NTD‐derived species. Consequently, tag effects should be considered when interpreting the aggregation behavior of such assembly‐prone domains.

#### 3.7.2 Metal‐Coordination‐Driven Engineered Condensates

*Dominant Tag‐Effect Types: V*

His‐tags are frequently employed as purification handles; however, they can mediate interactions with environmental metal ions, thereby altering protein aggregation and phase behavior. This phenomenon was demonstrated using an engineered construct comprising a proline‐rich motif (PRM), a Src homology 3 (SH3) domain, and a His‐tag (PRM‐SH3‐His), which was observed to undergo LLPS in response to divalent metal ions [25]. Specific transition metals (e.g., Ni^2+^, Zn^2+^, Cu^2+^, and Co^2+^) induced droplet formation via hexahistidine coordination; conversely, tag removal or EDTA treatment prevented this assembly. Furthermore, the specific metal identity and metal‐to‐protein ratio influenced the material properties of the condensates, including the critical concentration, internal protein mobility, and resistance to dissolution. Although this system was intentionally designed, these findings highlight an interpretational caveat: under permissive conditions, His‐tags can introduce artificial multivalency and promote condensate formation via metal coordination.

#### 3.7.3 BPTI (SCP Tags)

*Dominant Tag‐Effect Types: III/IV*

Bovine pancreatic trypsin inhibitor (BPTI) variants exemplify how tags serve as explicit design elements to control protein solubility and association, rather than acting as unintended confounding factors. In this system, short solubility‐controlling peptide (SCP) tags adjust physical properties without substantially compromising protein structure or activity. For example, attaching charged SCP tags containing lysine or arginine residues at the C‐terminus of a low‐solubility BPTI variant increased protein solubility by 4.2‐ to 6.2‐fold while preserving native folding and function, an effect consistent with electrostatic modulation of intermolecular association [133]. In contrast, introducing highly hydrophobic SCP tags enriched in isoleucine or leucine induced the formation of sub‐visible amorphous aggregates. Hydrophobic C‐terminal tags composed of five Leu or five Ile residues (C5L and C5I) increased the hydrodynamic radius from approximately 1.37 ± 0.05 nm for untagged BPTI‐19A to a range of 2.13 ± 0.23 to 3.81 ± 0.26 nm, as measured by dynamic light scattering. Static light scattering measurements also supported an increase in particle size, while circular dichroism (CD) spectroscopy indicated that the secondary structure remained largely intact, distinguishing these assemblies from amyloid fibrils [134]. Thus, the BPTI system demonstrates that short tags can stepwise modulate the propensity for protein association. This controlled modulation translates into functional applications; for instance, nanometer‐scale BPTI aggregates generated via SCP tags enhance the immune response in vivo [135]. In this context, SCP tags do not cause irreversible insolubilization but rather control aggregate size to achieve targeted functional effects, such as enhanced immunogenicity. This design strategy extends beyond BPTI, as similar SCP tag‐mediated control of association and immunogenicity has been applied to dengue virus envelope protein domain 3 (ED3) [136].

#### 3.7.4 SAK, HSC70, TNF‐α, EB1

*Dominant Tag‐Effect Type: III*

Fusion tags can alter the intrinsic biochemical and biophysical properties of proteins, including their structural stability, enzymatic activity, and oligomeric state distribution. For staphylokinase (SAK), the addition of an N‐terminal His‐tag shifted its pH‐dependent structural stability and activity [137]. Removing this tag restored near‐native physical properties, demonstrating that the His‐tag directly perturbs the folded state. Similarly, appending an N‐terminal His‐tag to tumor necrosis factor‐α (TNF‐α) attenuated its biological activity, with the magnitude of reduction depending on the specific tag length and design [138]. Furthermore, in the molecular chaperone HSC70, a His‐tag altered the intrinsic oligomerization profile [139]. These observations indicate that even short affinity tags can confound the interpretation of experimental outputs, such as stability and activity profiles.

Beyond stability and homomeric assembly, tags complicate the evaluation of heteromeric interactions. In the microtubule‐binding protein EB1, an affinity tag altered the apparent affinity and binding stoichiometry with microtubules [140]. Consequently, interaction parameters derived from tagged EB1 constructs do not accurately reflect the intrinsic behavior of the untagged protein. Taken together, these cases demonstrate that small functional tags perturb multiple biochemical and biophysical parameters. Therefore, careful construct validation is required before attributing observed behaviors to the intrinsic properties of the native protein.

### 3.8 Conceptual Summary

Collectively, these diverse case studies indicate that the impact of protein tagging is highly context‐dependent. Because protein aggregation and LLPS are fundamentally driven by weak, multivalent interactions, they are highly sensitive to the varied physical and chemical perturbations introduced by exogenous tags. Certain generalizable trends can be deduced; for instance, bulky solubility tags such as MBP and SUMO typically act as steric suppressors of assembly, whereas small chemical fluorophores often redirect aggregation pathways to alter aggregate morphology. However, these rules are not absolute. For example, the commonly used His‐tag can either promote or inhibit amyloid fibril formation depending on its attachment site and the intrinsic properties of the host protein. Similarly, different fluorescent fusion proteins can exert opposing effects on LLPS, and even the same tag can behave differently depending on the fusion partner. Consequently, a priori prediction of tag‐induced artifacts remains challenging. Although identifying specific aggregation‐prone regions or dominant driving forces (e.g., anticipating the electrostatic interference of a His‐tag) allows for some rational countermeasures, absolute predictability is elusive. Ultimately, the manifestation and magnitude of tagging effects are not dictated solely by the intrinsic properties of the tag itself, but rather by the delicate thermodynamic and structural balance among the tag, the target protein, and their specific environment.

## 4 Experimental Design for Disentangling Tag Effects

**Figure 3:**
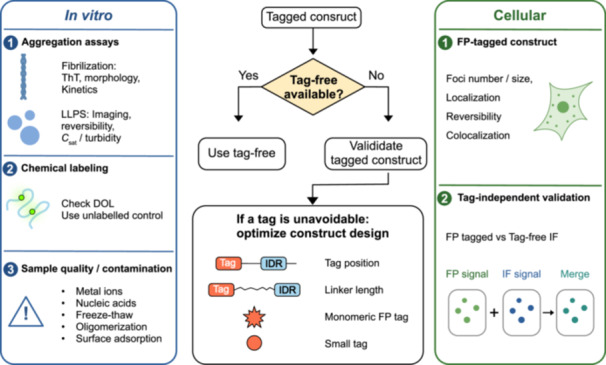
Experimental design for disentangling tag effects. The workflow summarizes key controls for distinguishing intrinsic protein assembly from tag‐ or construct‐dependent artifacts. Ideal designs use tag‐free proteins when feasible; otherwise, robust controls compare tagged constructs with tag‐cleaved or minimally modified counterparts under matched conditions. If a tag is unavoidable, experimental design must account for tag position, linker length, tag size, and fluorescent protein oligomeric state. In vitro assays should combine bulk readouts with morphology, kinetics, reversibility, and phase‐boundary measurements, such as *C*_sat_ or turbidity. Chemical labeling requires assessment of the degree of labeling and unlabeled controls. Rigorous preparation also controls for sample‐quality factors, including freeze–thaw history, adsorption, residual metal ions, nucleic acids, and pre‐existing oligomers. Cellular assays should validate fluorescent protein‐tagged phenotypes by tag‐independent detection or alternative construct designs. IF, immunofluorescence.

The presence of a tag can influence not only the propensity for self‐assembly but also its kinetics, concentration threshold, maturation trajectory, final morphology, and resulting cellular phenotypes. Consequently, a simple binary comparison between a tagged construct and an untagged control is often insufficient. Experimental designs should instead be tailored to determine which specific stage of the assembly process is altered. Furthermore, observed deviations may arise not solely from the tag itself, but also from its position, the linker sequence, residual scars after enzymatic cleavage, purification‐derived carryover, labeling stoichiometry, or sample handling history. This section outlines the minimal controls and complementary analytical assays required to assess different assembly modes, along with the primary confounding factors that should be rigorously excluded before attributing any behavioral change strictly to the tag. These considerations can be organized as a practical workflow for evaluating tag‐induced artifacts in aggregation and LLPS assays (Figure 3).

### 4.1 Assessing Tag Effects in Different Assembly Contexts

#### 4.1.1 In Vitro Aggregation and Fibrillization

For in vitro amyloid fibrillization assays, it is essential to establish a completely untagged construct as the primary reference state. If purifying an untagged protein proves technically prohibitive due to low solubility or yield, tagged constructs should be rigorously compared with their proteolytically cleaved counterparts under identical conditions. However, proper construct matching alone remains insufficient without the application of appropriate analytical techniques. Relying exclusively on fluorescent probes, such as ThT, frequently obscures tag‐induced partitioning into non‐fibrillar oligomers or amorphous aggregates and lacks the spatial resolution to detect structural polymorphism among mature fibrils. Consequently, these bulk kinetic measurements should be systematically complemented by orthogonal, high‐resolution morphological and structural assays, including TEM, AFM, CD, or sedimentation analyses. This multiparametric approach is necessary to accurately distinguish whether a tag changes the intrinsic fibrillization kinetics, redirects the assembly pathway, or alters the final fibril architecture [12, 15, 35, 36].

#### 4.1.2 LLPS Threshold and Material Properties

In LLPS studies, merely confirming condensate formation is insufficient because tags can alter the underlying phase behavior. Specifically, they can shift the critical concentration and salt dependency required for phase separation and perturb the internal fluidity of the resulting assemblies. Accurate assessment of condensate material properties, such as liquid‐ versus solid‐like states, and monitoring of time‐dependent hardening necessitate a direct comparison between tagged and tag‐cleaved samples under matched conditions. Beyond static imaging, dynamic properties should be evaluated using functional assays, such as droplet fusion, FRAP, temperature reversibility, and sensitivity to 1,6‐hexanediol (1,6‐HD). Finally, when fluorescent probes are employed to monitor these assembly processes, fluorophores themselves should be considered as additional potential sources of perturbation [13, 43].

#### 4.1.3 Intracellular Aggregates and Condensates

In cellular experiments, fluorescent foci formed by tagged proteins should not be interpreted in isolation as evidence for native aggregate or condensate formation, because the fluorescent tags themselves can promote, suppress, or reshape intracellular assembly. Therefore, whenever possible, tagged constructs should be compared with untagged or tag‐independently detected samples, such as those analyzed by immunostaining against the protein of interest. Additionally, different fluorescent tags and fusion designs should be tested because tag‐dependent artifacts depend strongly on the specific construct design. The observed structures should then be characterized along independent experimental axes. Specifically, material properties can be assessed by evaluating reversibility (e.g., via FRAP or chemical dissolution assays), while cellular identity can be determined through colocalization with established marker proteins [13, 33, 111].

#### 4.1.4 Fluorescent and Chemical Labeling

Chemical labels and fluorescent probes are smaller than fusion tags, but they can still influence protein assembly and phase behavior. Variations in labeling density or conjugation site can alter nucleation, partitioning, fibril growth, or surface interactions. Therefore, the behavior of labeled proteins should be directly compared with that of unlabeled controls, and the degree of labeling should be quantified. In general, labeled proteins are best treated as visualization tools; mechanistic and kinetic conclusions should be validated against the behavior of unlabeled counterparts [35, 36].

### 4.2 Major Confounders to Exclude before Assigning a Tag Effect

Before attributing observed differences in assembly behavior to the tag itself, alternative explanations related to construct design, sample preparation, and the assay context should be excluded. In vitro aggregation is often influenced by external factors such as nucleic acids, metal ions, ATP, and pH fluctuations [25, 93, 141, 142]. Accordingly, when the objective is to define how these variables affect the assembly process, untagged or rigorously tag‐cleaved proteins are preferable because tags can obscure the intrinsic response of the protein. In practice, apparent tag‐dependent effects may instead arise from differences in the tag position, linker design, residual protease cleavage scars, or uncleaved fractions, as well as from purification‐derived carryover such as metal ions, nucleic acids, free tags, or residual proteases [43, 53, 63, 143]. Residual metal ions after immobilized metal affinity chromatography (IMAC) can promote IDR oligomerization or aggregation; thus, EDTA treatment or thorough buffer exchange after purification may help exclude metal‐dependent artifacts. When a tag is placed close to an aggregation‐prone region, the assembly behavior may become sensitive to the tag position. In such cases, incorporating a longer linker may reduce direct coupling between the tag and the assembly‐prone segment. Final buffer composition, additives, concentration procedures, freeze‐thaw history, and surface adsorption can also alter assembly behavior independently of the tag [16, 124].

Therefore, observed differences should be interpreted as tag‐specific effects only after these experimental parameters are standardized between samples. If a discrepancy remains, structural approaches such as nuclear magnetic resonance (NMR) spectroscopy or cryo‐EM can help determine whether the tag directly alters intermolecular contacts, conformational dynamics, or the macromolecular architecture of the final assembly [21, 122].

## 5 Conclusion

Protein tags are not passive technical aids for purification, detection, or imaging; rather, they influence protein self‐assembly. Beyond simply modulating the propensity for aggregation or LLPS, tags alter nucleation kinetics, environmental thresholds, maturation pathways, and final morphological states. These tag‐induced perturbations are highly context‐dependent, varying with tag identity, fusion position, linker architecture, and the specific assembly types and aggregation parameters being analyzed. As illustrated by our framework, tag‐induced perturbations cannot be reduced to a single mechanism; rather, they must be evaluated as a sum of overlapping physical and chemical layers (Types I–VI).

Although highly stable globular proteins may tolerate tagging with minimal structural disruption, this tolerance is specific to the particular target protein and experimental condition, and does not indicate universal tag neutrality. Consequently, data derived from tagged constructs, while useful for initial discovery and cellular visualization, are frequently insufficient for drawing definitive mechanistic conclusions. This limitation in interpretation is particularly critical for IDPs and assembly‐prone domains when defining precise assembly pathways, resolving the architecture of final assembled states, or establishing causal links between these assemblies and biological functions. To ensure that observed phenomena are biologically relevant, experimental designs should incorporate untagged validation or, at a minimum, employ complementary construct variations—such as testing both N‐ and C‐terminal fusions or using cleavable linkers—to isolate tag‐dependent artifacts. Ultimately, protein tagging should be treated as a physical variable that actively shapes the assembly state, rather than being dismissed as a minor methodological necessity.

## Conflicts of Interest

The authors declare no conflicts of interest.

## Data Availability

Data sharing not applicable to this article as no datasets were generated or analyzed during the current study.
